# 4-Isopropyl-2,6-bis(1-phenylethyl)aniline 1, an Analogue of KTH-13 Isolated from* Cordyceps bassiana*, Inhibits the NF-*κ*B-Mediated Inflammatory Response

**DOI:** 10.1155/2015/143025

**Published:** 2015-12-27

**Authors:** Woo Seok Yang, Zubair Ahmed Ratan, Gihyeon Kim, Yunmi Lee, Mi-Yeon Kim, Jong-Hoon Kim, Jae Youl Cho

**Affiliations:** ^1^Department of Genetic Engineering, Sungkyunkwan University, Suwon 440-746, Republic of Korea; ^2^Department of Pediatrics, Institute of Child and Maternal Health (ICMH), Matuail, Dhaka 1362, Bangladesh; ^3^Department of Chemistry, Kwangwoon University, Seoul 139-701, Republic of Korea; ^4^School of Systems Biological Science, Soongsil University, Seoul 156-743, Republic of Korea; ^5^Department of Physiology, College of Veterinary Medicine, Chonbuk National University, 79 Gobong-ro, Iksan 54596, Republic of Korea

## Abstract

The* Cordyceps* species has been a good source of compounds with anticancer and anti-inflammatory activities. Recently, we reported a novel compound (4-isopropyl-2,6-bis(1-phenylethyl)phenol, KTH-13) with anticancer activity isolated from* Cordyceps bassiana* and created several derivatives to increase its pharmacological activity. In this study, we tested one of the KTH-013 derivatives, 4-isopropyl-2,6-bis(1-phenylethyl)aniline 1 (KTH-13-AD1), with regard to anti-inflammatory activity under macrophage-mediated inflammatory conditions. KTH-13-AD1 clearly suppressed the production of nitric oxide (NO) and reactive oxygen species (ROS) in lipopolysaccharide (LPS) and sodium nitroprusside- (SNP-) treated macrophage-like cells (RAW264.7 cells). Similarly, this compound also reduced mRNA expression of inducible NO synthase (iNOS) and tumor necrosis factor-*α* (TNF-*α*), as analyzed by RT-PCR and real-time PCR. Interestingly, KTH-13-AD1 strongly diminished NF-*κ*B-mediated luciferase activities and nuclear translocation of NF-*κ*B family proteins. In accordance, KTH-13-AD1 suppressed the upstream signaling pathway of NF-*κ*B activation, including I*κ*B*α*, IKK*α*/*β*, AKT, p85/PI3K, and Src in a time- and dose-dependent manner. The autophosphorylation of Src and NF-*κ*B observed during the overexpression of Src was also suppressed by KTH-13-AD1. These results strongly suggest that KTH-13-AD1 has strong anti-inflammatory features mediated by suppression of the Src/NF-*κ*B regulatory loop.

## 1. Introduction

Under innate immune conditions, the inflammatory responses mediated by macrophages, mast cells, and neutrophils comprise an important barrier against infectious pathogens, such as viruses, fungi, and bacteria, as well as chemical toxins [1, 2]. Among the cellular components of innate immunity, macrophages are regarded as central inflammatory cells, as they identify external pathogens using special surface receptors (e.g., toll-like receptors (TLRs)) and are widely distributed in the human body. The inflammatory responses mediated by macrophages and their role in pathophysiology have been previously studied [3]. Macrophages are activated by lipopolysaccharide (LPS) via its counterreceptor, TLR4. Activated macrophages induce various intracellular signaling cascades, including Src, Syk, phosphatidylinositide 3-kinase (PI3K), Akt, inhibitor of *κ*B (I*κ*B) kinase (IKK) *αβ*, and I*κ*B [4–6]. The signaling pathway also stimulates the nuclear translocation of nuclear factor- (NF-) *κ*B and activator protein AP-1, triggering the expression of inflammatory genes that lead to secretion of inflammatory mediators (e.g., nitric oxide (NO), reactive oxygen species (ROS), prostaglandin E_2_ (PGE_2_), chemokines, and cytokines (e.g., tumor necrosis factor- (TNF-) *α*)) [7–9]. Recently, ample evidence has suggested that unchecked, prolonged inflammatory responses can cause serious immunological diseases, including diabetes, septic shock, cancer, arthritis, and cardiovascular disease. The understanding of inflammatory responses and exploration of strategies for suppressing inflammation are thus considered appropriate approaches to reducing disease incidence [10–13].

The* Cordyceps* genus including* Cordyceps sinensis*,* Cordyceps militaris*,* Cordyceps pruinosa*, and* Cordyceps bassiana* grow in Korea, Japan, China, and the Congo. The* Cordyceps* genus can be administered through traditional routes and is known to ameliorate various inflammatory diseases, including chronicle bronchitis, asthma, and eczema. The biological and pharmacological activities of* Cordyceps* genus are antioxidative, antiviral, antifibrotic, anti-inflammatory, antinociceptive, antiangiogenic, antiplatelet aggregation, and antidiabetic [14, 15]. Studies have also demonstrated the anti-inflammatory mechanisms of butanol (BF) and hexane (HF) fractions of* Cordyceps bassiana* [16]. However, the specific chemical compounds responsible for the plant's anti-inflammatory properties have not yet been elucidated. Recently, we isolated a promising novel compound [KTH-13: 4-isopropyl-2,6-bis(1-phenylethyl)phenol] with anticancer activity from* Cordyceps bassiana* [17]. Despite the novel chemical structure of this compound, we have established a method for its total synthesis and derivatization to develop more effective molecules. So far, almost 60 compounds were newly synthesized and tested to check their activities by employing NO assay and antiproliferative activity. Of them, interestingly, KTH-13-amine-diastereomer 1 [4-isopropyl-2,6-bis(1-phenylethyl)aniline 1 (KTH-13-AD1)] has been reported to have stronger activity than that of the original compound in terms of anticancer activity (data not shown). In this study, therefore, we further aimed to demonstrate the anti-inflammatory potential of KTH-13-AD1, a derivative of KTH-13, and to explore its mechanism of action using activated macrophages.

## 2. Materials and Methods

### 2.1. Materials

Sodium nitroprusside (SNP), 3-(4,5-dimethylthiazol-2-yl)-2,5-diphenyltetrazolium bromide (MTT), dihydrorhodamine 123 (DHR123), fluorescein isothiocyanate- (FITC-) dextran, ascorbic acid, and LPS (*E. coli* 0111:B4) were purchased from Sigma Chemical Co. (St. Louis, MO, USA). Fetal bovine serum and RPMI 1640 were obtained from Gibco (Grand Island, NY, USA). The murine macrophage cell line RAW264.7 and human embryonic kidney (HEK) 293 cells were purchased from the American Type Culture Collection (Rockville, MD, USA). PP2 was obtained from Calbiochem (La Jolla, CA, USA). Luciferase constructs containing binding promoters for NF-*κ*B and AP-1 were gifts from Professors Chung, Hae Young (Pusan National University, Pusan, Korea) and Rhee, Man Hee (Kyungpook National University, Daegu, Korea). Phospho- and total protein-specific antibodies to p65, p50, c-Fos, c-Jun, I*κ*B*α*, IKK*β*, AKT, p85, Src, Syk, lamin A/C, and *β*-actin were obtained from Cell Signaling Technology (Beverly, MA, USA). Primers (Table 1) designed in our laboratory were synthesized by Bioneer (Daejeon, Korea).

### 2.2. Preparation of KTH-13-AD1

**Figure 1:**
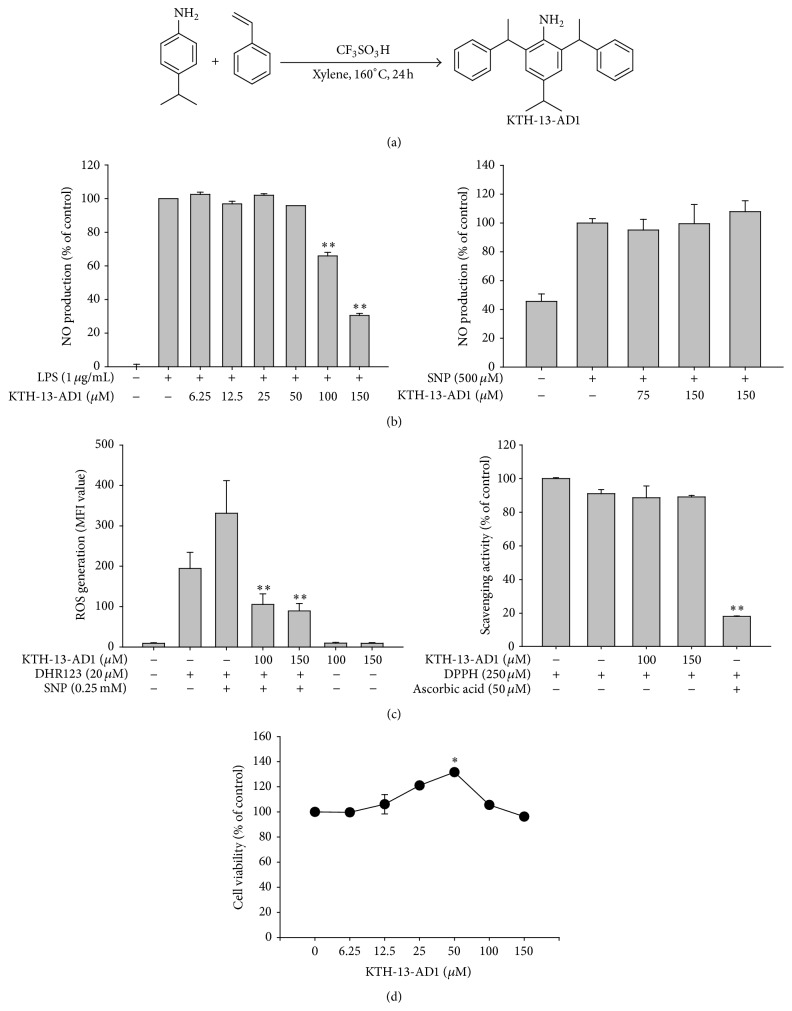
Effects of KTH-13-AD1 on macrophage-mediated inflammatory responses. (a) Chemical structure of KTH-13-AD1. (b) NO production in LPS-treated RAW264.7 cells or induced by sodium nitroprusside (SNP), as determined by Griess assay. ((c), left panel) The ROS scavenging effect of KTH-13-AD1 was measured with SNP (0.25 mM)-treated RAW264.7 cells, using DHR123 (20 *μ*M). The level of ROS was determined through flow cytometric analysis. ((c), right panel) The radical scavenging activity of KTH-13-AD1 was determined using DPPH (250 *μ*M) assay. (d) The effect of KTH-13-AD1 on the viability of RAW264.7 cells was evaluated using MTT assay. ^*∗*^ *P* < 0.05 and ^*∗∗*^ *P* < 0.01 compared to the vehicle control or normal group.

To synthesize KTH-13-AD1 (Figure 1(a)), a solution of 4-isopropylaniline (4.00 g, 29.6 mmol) in xylene (14 mL) was mixed with styrene (9.48 g, 91.1 mmol) and CF_3_SO_3_H (1.0 mL, 11.4 mmol). The reaction mixture was allowed to heat to 160°C and stirred for 24 h. At that time, the reaction was allowed to cool to room temperature and the volatiles were removed under* vacuo*. The resulting residue was purified by silica gel column chromatography (hexanes : EtOAc = 9 : 1) to afford the desired KTH-13-AD1 (2.60 g, 7.57 mmol, and 1 : 1 diastereomers) in 26% yield as a brown oil. Infrared (IR) spectra of KTH-13-AD1 were recorded on a Bruker Vertex 70 spectrophotometer, *ν*_max⁡_ in cm^−1^. Bands are characterized as strong (s), medium (m), and weak (w). ^1^H Nuclear magnetic resonance (NMR) spectra of this compound were recorded on a JEOL JNM-AL400 (400 MHz) spectrometer. Chemical shifts are reported in ppm from tetramethylsilane with the solvent resonance as the internal standard (CDCl_3_: *δ* 7.27 ppm). Data are reported as follows: chemical shift, multiplicity (s = singlet, d = doublet, t = triplet, q = quartet, and m = multiplet), coupling constants (Hz), and integration. ^13^C NMR spectra were recorded on a JEOL JNM-AL400 (100 MHz) spectrometer with complete proton decoupling. Chemical shifts are reported in ppm from tetramethylsilane with the solvent resonance as the internal standard (CDCl_3_: *δ* 77.16 ppm). Low-resolution mass spectrometry was performed on an Agilent 6890N GC (Hewlett-Packard Co., Palo Alto, California, USA).

^1^H NMR (400 MHz, CDCl_3_, diastereomer 1): *δ* 7.40–7.12 (m, 12H), 4.07 (q,* J* = 6.9 Hz, 2H), 3.29 (br s, 2H), 3.00 (sept,* J* = 6.9 Hz, 1H), 1.67 (d,* J* = 7.1 Hz, 6H), 1.37 (d,* J* = 7.1 Hz, 6H); ^13^C NMR (100 MHz, CDCl_3_): *δ* 145.8, 139.4, 138.9, 130.2, 128.7, 127.5, 126.3, 123.5, 40.2, 33.7, 24.4, 22,2; IR (neat): 3471 (s), 3384 (s), 3060 (m), 3024 (m), 2982 (s), 2869 (s), 2293 (w), 1947 (w), 1878 (w), 1803 (w), 1623 (s), 1600 (s), 1471 (s), 1450 (s), 1373 (m), 1318 (m), 1257 (m), 1172 (m), 1027 (m), 881 (s), 761 (s), 700 (s) cm^−1^; LR-MS (ESI):* m/z* calculated for C_25_H_30_N ([M + H]^+^) 344.2, and found 344.2.

### 2.3. Cell Culture

RAW264.7 and HEK293 cells were cultured with RPMI1640 medium supplemented with 10% heat-inactivated FBS, glutamine, and antibiotics (penicillin and streptomycin) at 37°C in a 5% CO_2_ atmosphere. In each experiment, cells were detached with a scraper. Examination of cell densities at 2 × 10^6^ cells/mL revealed that the proportion of dead cells was consistently <1% according to trypan blue dye exclusion as the criterion for viability.

### 2.4. NO Production

RAW264.7 macrophage cells (1 × 10^6^ cells/mL) were cultured for 18 h, pretreated with KTH-13-AD1 (0 to 200 *μ*M) for 30 min, and further incubated with LPS (1 *μ*g/mL) for 24 h. The inhibitory effect of KTH-13-AD1 on LPS-induced NO production was determined by analyzing NO level using Griess reagent, as previously described [18, 19]. The OD at 550 nm (OD_550_) was measured using a SpectraMax 250 microplate reader (Molecular Devices, Sunnyvale, CA, USA).

### 2.5. Determination of Reactive Oxygen Species Generation

The level of intracellular ROS was determined by recording the change in fluorescence resulting from the oxidation of the fluorescent probe DHR123. Briefly, 5 × 10^5^ RAW264.7 cells were exposed to KTH-13-AD1 (0 to 150 *μ*M) for 30 min and then incubated with SNP (0.25 mM) at 37°C for 20 min to induce ROS production. The cells were further incubated with 20 *μ*M of the fluorescent probe DHR123 for 30 min at 37°C. The degree of fluorescence, which corresponded to the level of intracellular ROS, was determined using a FACScan flow cytometer (Becton-Dickinson), as reported previously [20].

### 2.6. Measurement of Phagocytic Uptake

To measure the phagocytic activity of RAW264.7 cells, we modified a previously reported method [21]. RAW264.7 cells (5 × 10^4^) were pretreated with KTH-13-AD1 (0 to 150 *μ*M) for 1 h, resuspended in 100 *μ*L phosphate buffered saline (PBS) containing 1% human AB serum, and then incubated with FITC-dextran (1 mg/mL) at 37°C for 20 min. The reactions were stopped by adding 2 mL ice-cold PBS containing 1% human serum and 0.02% sodium azide. The cells were then washed three times with cold PBS-azide and analyzed on a FACScan flow cytometer (Becton-Dickinson, San Jose, CA, USA), as reported previously [20].

### 2.7. Flow Cytometric Analysis

The level of FITC-dextran or ROS in RAW264.7 cells was determined through flow cytometric analysis [22, 23]. RAW264.7 cells (2 × 10^6^ cells/mL) treated with KTH-13-AD1 in the presence or absence of FITC-dextran (1 mg/mL) or DHR123 (20 *μ*M) were washed with staining buffer containing 2% rabbit serum and 1% sodium azide in PBS and then incubated with direct-labeled antibodies for an additional 45 min on ice. After washing three times with staining buffer, stained cells were analyzed on a FACScan flow cytometer (Becton-Dickinson).

### 2.8. Cell Viability Test

RAW264.7 cells (1 × 10^6^ cells/mL) were cultured for 18 h, after which KTH-13-AD1 (0 to 150 *μ*M) was added for the final 24 or 8 h of culture. The cytotoxic effects of ATS-E3 KTH-13-AD1 were then evaluated using a conventional MTT assay, as reported previously [24, 25]. For the final 3 h of culture, 10 *μ*L MTT solution (10 mg/mL in PBS, pH 7.4) was added to each well. The incubation was stopped by the addition of 15% sodium dodecyl sulfate (SDS) to each well, which solubilized the formazan [26]. The absorbance at 570 nm (OD_570–630_) was measured using a SpectraMax 250 microplate reader (BioTek, Bad Friedrichshall, Germany).

### 2.9. Analysis of mRNA Expression Using Reverse Transcription-Polymerase Chain Reaction (RT-PCR)

RAW264.7 cells (1 × 10^6^ cells/mL) were cultured for 18 h, pretreated with KTH-13-AD1 (0 to 150 *μ*M) for 30 min, and further cultured with LPS (1 *μ*g/mL) for 6 h. The inhibitory effect of KTH-13-AD1 on the expression of iNOS and TNF-*α* was determined using semiquantitative RT-PCR and real-time quantitative reverse transcription-polymerase chain reaction (qRT-PCR), as reported previously [18, 27]. The primers (Bioneer, Daejeon, Korea) used in these reactions are listed in Table 1.

### 2.10. Plasmid Transfection and Luciferase Reporter Gene Activity Assay

HEK293 cells (1 × 10^6^ cells/mL) were transfected with 1 *μ*g plasmids to drive the expression of *β*-galactosidase and either NF-*κ*B-Luc or AP-1-Luc in the presence or absence of an inducing molecule (MyD88, TRIF, or HA-Src). Transfections were performed using the PEI method in 12-well plates, as previously outlined [28, 29]. Transfected cells were used at 48 h after transfection in all experiments. Cells were treated with KTH-13-AD1 for the final 8 h of each experiment. Luciferase assays were performed using the Luciferase Assay System (Promega, Madison, WI, USA), as previously reported [30].

### 2.11. Preparation of Cell Lysates and Immunoblotting Analysis

RAW264.7 cells (5 × 10^6^ cells/mL) were washed three times in cold PBS supplemented with 1 mM sodium orthovanadate, resuspended in lysis buffer (20 mM Tris-HCl, pH 7.4, 2 mM EDTA, 2 mM ethyleneglycotetraacetic acid, 50 mM *β*-glycerophosphate, 1 mM sodium orthovanadate, 1 mM dithiothreitol, 1% Triton X-100, 10% glycerol, 10 *μ*g/mL aprotinin, 10 *μ*g/mL pepstatin, 1 mM benzamide, and 2 mM PMSF), lysed by sonication, and rotated for 30 min at 4°C. The lysates were clarified by centrifugation at 16,000 ×g for 10 min at 4°C and stored at −20°C until use. The soluble fractions of the cell lysates were immunoblotted, and total and phosphoprotein levels of c-Fos, c-Jun, p50, p65, I*κ*B*α*, IKK, AKT, p85, Src, Syk, lamin A/C, and *β*-actin were visualized, as previously reported [31].

### 2.12. Statistical Analysis

Data are expressed as the mean ± standard deviation (SD), as calculated from one (*n* = 6) of two independent experiments. Other data are representative of three different experiments with similar results. For statistical comparisons, the results were analyzed using analysis of variance/Scheffe's post hoc test and the Kruskal-Wallis/Mann-Whitney test. A *P* value <0.05 was considered to be statistically significant. All statistical tests were conducted using SPSS (SPSS Inc., Chicago, IL, USA).

## 3. Results and Discussion

In previous studies, we and other investigators have reported on the anti-inflammatory, anticancer, and immunomodulatory activities of* Cordyceps bassiana* [32–35]. Unlike other* Cordyceps* species, such as* Cordyceps militaris* and* Cordyceps sinensis*, few studies have been conducted on the active components of* Cordyceps bassiana*. Recently, we identified a promising new compound called 4-isopropyl-2,6-bis(1-phenylethyl)phenol with anticancer activity against several cancer cell lines, such as C6 glioma and MDA-MB-231 [33]. We further established a method for total synthesis of this compound in order to facilitate its mass production and derivatization. In this study, we tested one (KTH-13-AD1 (Figure 1(a))) of its derivatives, specifically to see whether this compound is able to suppress macrophage-mediated inflammatory responses.

As shown in Figure 1(b), left panel, the production of NO was reduced by up to 70% in a dose-dependent manner by KTH-13-AD1 in LPS-stimulated macrophage-like RAW264.6 cells at 150 *μ*M, though it did not downregulate NO released from SNP, a drug that directly releases NO [36] in vitro (Figure 1(b), right panel). These results indicate that KTH-13-AD1 does not act as a chemically directed neutralizing agent but rather as a modulator of NO biosynthesis during TLR4 stimulation following LPS treatment. In similar fashion, the radical scavenging activity of KTH-13-AD1 was observed only at 100 and 150 *μ*M when applied to SNP-treated cells (Figure 1(c), left panel) but not in the DPPH assay (Figure 1(c), right panel). Since KTH-13-AD1 did not suppress the viability of RAW264.7 cells (Figure 1(d)), the results demonstrate that though this compound does not have direct antioxidative activity, it displays anti-inflammatory properties that indirectly suppress the production of inflammatory mediators, such as NO, similarly to ginsenosides that have only anti-inflammatory properties [37, 38]. However, unlike these compounds, flavonoids (e.g., quercetin, luteolin, and kaempferol) and anthraquinones (e.g., rhodoptilometrin), are active components of numerous medicinal plants and have been reported to demonstrate both antioxidative and anti-inflammatory activities [39–42].

**Figure 2:**
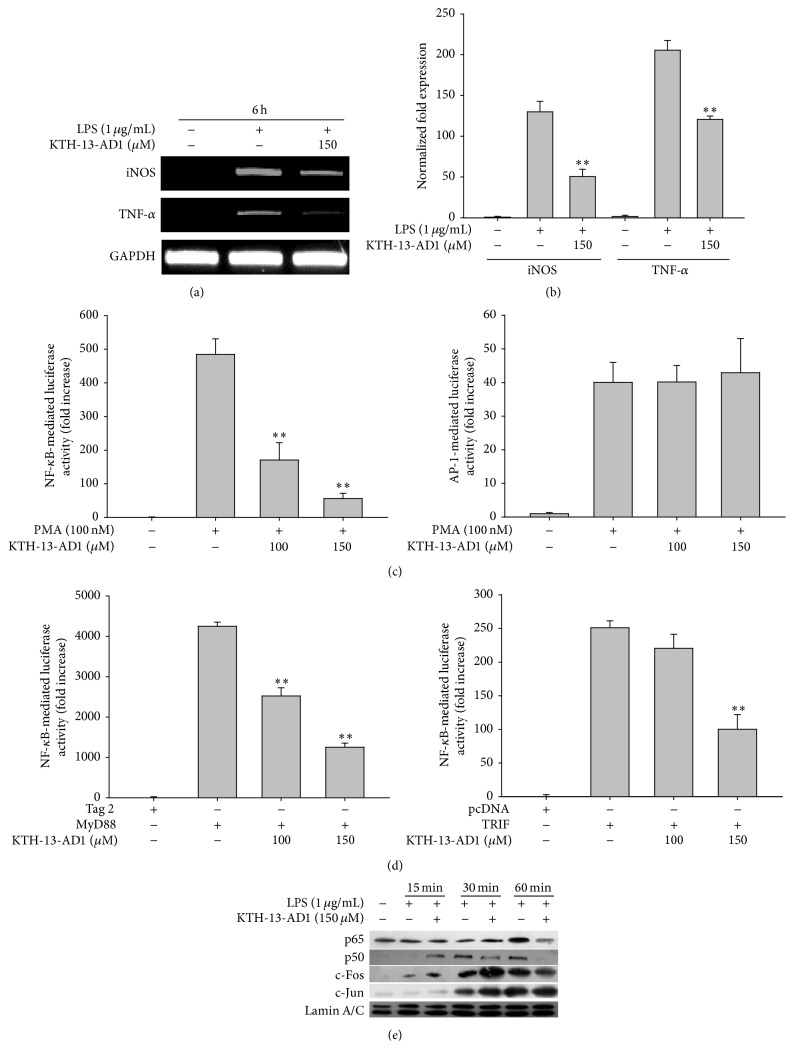
Effects of KTH-13-AD1 on transcriptional activation of LPS-mediated inflammatory responses. (a, b) mRNA levels of proinflammatory genes (iNOS and TNF-*α*) in LPS-treated RAW264.7 cells pretreated with KTH-13-AD1 (150 *μ*M), as analyzed by RT-PCR (a) and real-time PCR (b). (c, d) Transcriptional activation of inflammatory responses was examined using a luciferase reporter gene assay with HEK293 cells transfected with NF-*κ*B-Luc or AP-1-Luc, as well as with *β*-gal (as a transfection control) under either treatment with PMA or cotransfection with FLAG-MyD88 or CFP-TRIF (1 *μ*g/mL each). Luciferase activity was measured using a luminometer. (e) The nuclear translocation levels of AP-1 (c-Jun and c-Fos) and the NF-*κ*B (p65 and p50) family in LPS-treated RAW264.7 cells (5 × 10^6^ cells/mL) pretreated with KTH-13-AD1, as examined using nuclear fractionation and immunoblotting analysis. ^*∗∗*^ *P* < 0.01 compared to the vehicle control.

To determine the mechanism by which KTH-13-AD1 suppresses NO production, we investigated TLR4-mediated transcriptional activation levels in LPS-treated RAW264.7 cells pretreated with KTH-13-AD1. First, we evaluated mRNA levels in proinflammatory genes, including iNOS and TNF-*α*, using quantitative real-time and semiquantitative PCR. As shown in Figures 2(a) and 2(b), KTH-13-AD1 (150 *μ*M) significantly decreased the mRNA levels of iNOS and TNF-*α*, suggesting that KTH-13-AD1-driven suppression of inflammatory responses occur at the transcriptional level. Since NF-*κ*B and AP-1 are representative of transcription factors regulating inflammatory responses [43, 44], we determined the ability of this compound to suppress the activation of NF-*κ*B and AP-1 using a luciferase reporter gene assay with promoter sites for NF-*κ*B and AP-1. To determine how KTH-13-AD1 regulates promoter activity in inflammatory genes, we pretreated the KTH-13-AD1 for 30 min before activating HEK293 cells with PMA, an activator of PKC that induces the inflammatory signaling pathway [45]. As shown in Figure 2(c), luciferase activity mediated by NF-*κ*B and AP-1 was greatly enhanced by PMA, up to 500- and 40-fold, respectively. Interestingly, KTH-13-AD1 (100 and 150 *μ*M) strongly suppressed NF-*κ*B-mediated luciferase activity (Figure 2(c), left panel) but not AP-1 (Figure 2(c), right panel). It has also been reported that MyD88 and TRIF, adaptor molecules regulating the TLR4-mediated intracellular signaling pathway [46], are good inducers of luciferase activity in HEK293 cells [47, 48]. The results in Figure 2(d) support this conclusion, as cotransfection of these molecules increased both NF-*κ*B- and AP-1-mediated luciferase activity up to 4,000- and 250-fold, respectively, as seen in previous studies [48]. Comparing these two conditions, MyD88-induced NF-*κ*B activation was strongly suppressed by KTH-13-AD1, with 100 *μ*M of the compound inhibiting activation by 45% (Figure 2(d)). In addition, KTH-13-AD1 reduced the nuclear translocation of the NF-*κ*B subunits p65 and p50 at 30 and 60 min, although there was no inhibition at 15 min (Figure 2(e)). These results suggest that the NF-*κ*B signaling pathway affecting the activation of NF-*κ*B dimerization at 30 to 60 min might be a potential target for KTH-13-AD1. These inhibitory patterns observed by KTH-13-AD1 treatment seem to also imply that NF-*κ*B activation signals might be timely regulated by some upstream signaling units.

**Figure 3:**
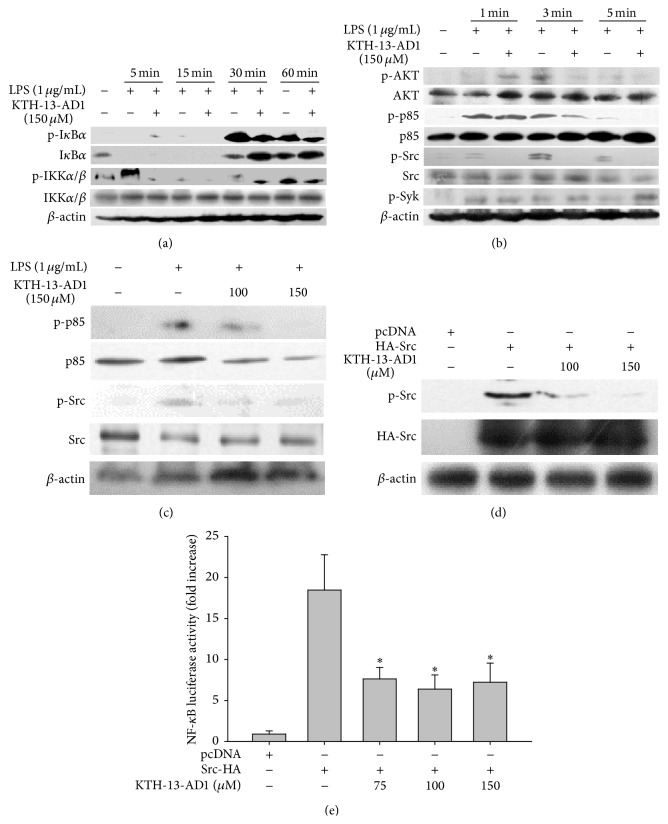
Effect of KTH-13-AD1 on the activation of upstream signaling cascades of NF-*κ*B. (a, b, c) Phospho- and total protein levels of I*κ*B*α*, IKK*α*/*β*, AKT, p85, Src, Syk, and *β*-actin from cell lysates were determined through immunoblotting analysis. (d) The inhibitory activity of KTH-13-AD1 on the phosphorylation of p85 and Src, triggered by HA-Src overexpression in HEK293 cells, was evaluated using immunoblotting analysis. (e) NF-*κ*B-mediated luciferase activity triggered by HA-Src overexpression in HEK293 cells, either in the presence or absence of KTH-13-AD1, was evaluated using luminometric analysis. ^*∗*^ *P* < 0.05 compared to the vehicle control.

To prove such raised possibility, we subsequently focused on identifying the NF-*κ*B regulatory pathways that are recognized by this compound. We further investigated the phosphorylation levels of upstream NF-*κ*B activation proteins. First, we determined the phosphorylation level of I*κ*B*α*, an essential step in the nuclear translocation of NF-*κ*B [49]. The phosphorylation of I*κ*B*α* was blocked by KTH-13-AD1 at 15, 30, and 60 min, while the total form of I*κ*B*α* was greatly increased at 30 and 60 min, compared to LPS treatment alone (Figure 3(a)), implying that this compound is able to suppress both phosphorylation and degradation of I*κ*B*α* between 30 min and 1 h. In addition, KTH-13-AD1 was found to reduce the phosphorylation of IKK*α*/*β*, upstream of I*κ*B*α*, at 5 min (Figure 3(a)). Similarly, the phosphorylation of AKT and p85/PI3K, upstream enzymes for IKK activation, was blocked by KTH-13-AD1 at 3 to 5 min (Figure 3(b)). Additionally, Src phosphorylation occurring at 1 and 3 min was also suppressed by this compound (Figure 3(b)). To confirm our results, we evaluated the dose-responsive activity of KTH-13-AD1 on the phosphorylation levels of putative target proteins, p85/PI3K and Src. As seen in Figure 3(c), the phosphorylation of p85 and Src was continuously diminished by KTH-13-AD1 (100 and 150 *μ*M) under the same conditions. As these results strongly suggest that the putative target of KTH-13-AD1 is Src, we next analyzed whether this compound is able to regulate the autophosphorylation of Src, using an overexpression strategy, as reported previously [50, 51]. As shown in Figure 3(d), KTH-13-AD1 (100 and 150 *μ*M) clearly decreased the level of Src phosphorylation, implying that Src is directly modulated by this compound. This is further supported by the fact that the promoter activity of NF-*κ*B, as stimulated by Src overexpression, was also blocked by KTH-13-AD1 (Figure 3(e)). Based on these results, we propose that Src serves as a potential target protein in KTH-13-AD1-mediated anti-inflammatory actions. Many anti-inflammatory compounds, both naturally occurring and chemically synthesized, have been found to possess direct inhibitory activity against Src kinase [50–52]. Additionally, previous studies have reported on Src activity under NF-*κ*B activation and inflammatory gene expression [51, 53, 54], suggesting an important role of Src in inflammatory responses and in oncogenic activity.

**Figure 4:**
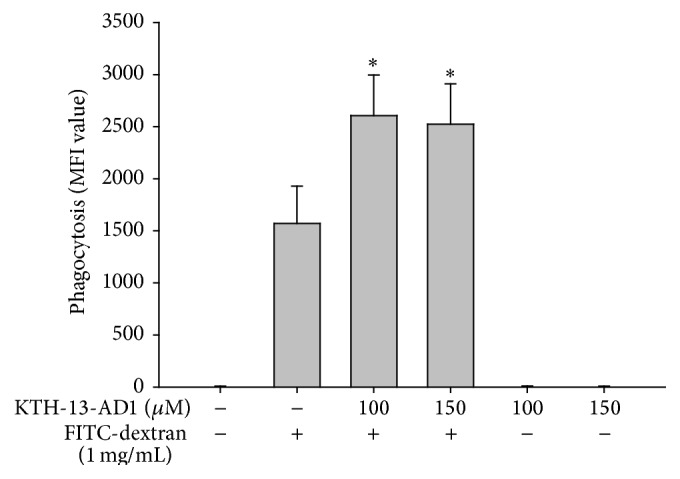
Effect of KTH-13-AD1 on the phagocytic uptake of RAW264.7 cells. RAW264.7 cells preincubated with KTH-13-AD1 were treated with FITC-dextran (1 mg/mL) for 20 min. The level of dextran uptake was determined through flow cytometric analysis. ^*∗*^ *P* < 0.05 compared to the vehicle control.

Finally, to demonstrate whether KTH-13-AD1 modulates macrophage phagocytic uptake, FITC-dextran was applied to RAW264.7 cells during exposure to this compound. As shown in Figure 4, there was no inhibition of phagocytosis by KTH-13-AD1, as measured by FITC-derived fluorescence [55, 56]; however, significant enhancement was observed at 100 and 150 *μ*M concentrations, implying that the compound stimulates macrophage phagocytosis, an important step in the innate immune response. This result suggests that innate immune responses might be regulated by KTH-13-AD1 via different cellular mechanisms.

**Figure 5:**
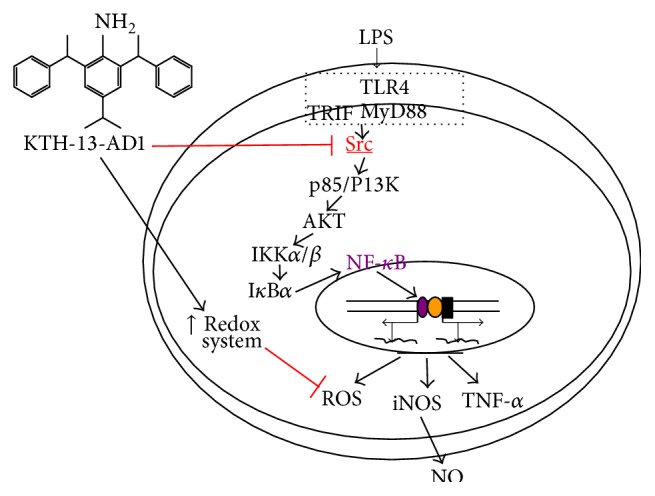
The putative inhibitory pathway of macrophage-mediated inflammatory signaling responses, as suppressed by KTH-13-AD1.

In summary, KTH-13-AD1 demonstrated impressive suppression of inflammatory mediator production, including ROS and NO, without inducing cytotoxicity, while this compound strongly enhanced the phagocytotic uptake, suggesting that KTH-13-AD1 is able to regulate cellular responses related to innate immunity. In addition, the transcriptional activation of NF-*κ*B and its upstream signaling pathway, composed of Src, p85/PI3K, and AKT, was blocked by KTH-13-AD1, as summarized in Figure 5. Thus, the present study proposes that KTH-13-AD1, a chemical analog of KTH-13 extracted from* Cordyceps bassiana*, has clinical utility as an anti-inflammatory drug. We will focus on analyzing this possibility in future studies.

## Figures and Tables

**Table 1:** Sequences of primers used in real-time PCR analysis.

Gene	Primer sequences
iNOS	
F	5′-CCCTTCCGAAGTTTCTGGCAGCAGC-3′
R	5′-GGCTGTCAGAGCCTCGTGGCTTTGG-3′
TNF-*α*	
F	5′-TTGACCTCAGCGCTGAGTTG-3′
R	5′-CCTGTAGCCCACGTCGTAGC-3′
GAPDH	
F	5′-CACTCACGGCAAATTCAACGGCAC-3′
R	5′-GACTCCACGACATACTCAGCAC-3′
